# NAP1L1 Functions as a Tumor Promoter *via* Recruiting Hepatoma-Derived Growth Factor/c-Jun Signal in Hepatocellular Carcinoma

**DOI:** 10.3389/fcell.2021.659680

**Published:** 2021-07-23

**Authors:** Ye-wei Zhang, Qian Chen, Bo Li, Hai-Yang Li, Xue-Ke Zhao, Yan-yi Xiao, Shu Liu, Shi Zuo

**Affiliations:** ^1^Department of Clinical Medicine, Guizhou Medical University, Guiyang, China; ^2^Department of Hepatobiliary Surgery, The Affiliated Hospital of Guizhou Medical University, Guiyang, China; ^3^Department of Infectious Diseases, The Affiliated Hospital of Guizhou Medical University, Guiyang, China; ^4^Department of Obstetrics and Gynecology, The Third Affiliated Hospital of Southern Medical University, Guangzhou, China; ^5^Department of Breast Surgery, The Affiliated Hospital of Guizhou Medical University, Guiyang, China

**Keywords:** hepatocellular carcinoma (HCC), HDGF, c-Jun, proliferation, NAP1L1

## Abstract

NAP1L1 has been reported to be significantly involved in the carcinogenesis of hepatocellular carcinoma (HCC). Yet, its detailed molecular basis is still to be determined. Based on the analysis of The Cancer Genome Atlas (TCGA) database, NAP1L1 mRNA was found to be upregulated and predicted the poor prognosis initially. Subsequently, consistent with the prediction, the upregulated expression of NAP1L1 mRNA and protein levels was confirmed by quantitative polymerase chain reaction (qPCR), Western blot, and immunohistochemistry assays. Upregulated NAP1L1 protein positively promoted the disease progression and poor prognosis of HCC. In addition, NAP1L1 protein expression was considered as an independent prognostic factor in HCC. Inhibition of NAP1L1 expression by siRNA or shRNA pathway significantly reduced the cell proliferation and cell cycle transformation *in vitro* and *in vivo*. Mechanism analysis first showed that the function of NAP1L1 was to recruit hepatoma-derived growth factor (HDGF), an oncogene candidate widely documented in tumors. Furthermore, the latter interacted with c-Jun, a key oncogenic transcription factor that can induce the expression of cell cycle factors and thus stimulate the cell growth in HCC. Finally, transfecting HDGF or c-Jun could reverse the suppressive effects on HCC growth in NAP1L1-suppressed HCC cells. Our data indicate that NAP1L1 is a potential oncogene and acts *via* recruiting HDGF/c-Jun in HCC.

## Introduction

Liver cancer is one of the leading causes of cancer-related deaths in the world (Akinyemiju et al., 2017), also causing the fourth deaths in most common malignancy and the third deaths in leading tumor-related deaths in China. Liver cancer is classified as primary and metastatic liver cancer. In China, hepatocellular carcinoma (HCC) accounts for about 85–90% of primary liver cancer (Zhou et al., 2018). Therefore, we focused on HCC in this study.

Hepatocellular carcinoma is a type of heterogeneous cancer with a lot of factors implicated in its development, with chronic infection and cirrhosis by hepatitis B virus (HBV) being the most prevalent (Beasley, 1988; McGlynn et al., 2015; Yamashita and Kaneko, 2016). Cirrhosis due to metabolic dysfunction, excessive alcohol consumption, non-alcoholic fatty liver disease (NAFLD), and hepatitis C virus (HCV) infection are also involved in HCC development (Venook et al., 2010; Morgan et al., 2013; Calzadilla and Adams, 2016; Yamashita and Kaneko, 2016). These factors alone or together lead to the imbalance of gene expression in the normal liver, inducing the occurrence and development of HCC (Lin et al., 2019, 2020; Liu et al., 2020).

NAP1L1 belongs to the human counterpart of the yeast NAP-I protein, a histone-binding factor involved in the cumulative nucleosome formation. NAP1L1 has been shown as a potential tumor promoter and participates in the pathogenesis of several tumors including colorectal cancer, renal cancer, and pancreatic neuroendocrine neoplasm (Schimmack et al., 2014; Zhai et al., 2018; Queiroz et al., 2020). Recent studies on HCC showed that PRDM8 suppresses the occurrence of tumor *via* interacting with NAP1L1 (Chen et al., 2018). Furthermore, NAP1L1 is modulated by the LncRNA CDKN2B-AS1/NAP1L1 axis and participates in the pathogenesis of HCC. However, the molecular basis of NAP1L1 in modulating HCC proliferation is still unclear (Huang et al., 2018).

Here, we found that the NAP1L1 protein level was significantly elevated in both HCC patients and HCC cell lines. It was an unfavorable factor, boosting the clinical progression and poor prognosis of HCC patients. Further, NAP1L1 was shown to be an oncogene that recruits hepatoma-derived growth factor (HDGF). The latter interacts with c-Jun, thus stimulating cell cycle signal transition and thus finally inducing HCC proliferation. The data indicate that NAP1L1 is a tumor promoter, significantly involved in the pathogenesis of HCC.

## Materials and Methods

### Bioinformatics Assay

BIOGRID web^1^ was used to find the potential biomarker interacting with NAP1L1 and HDGF. UALCAN web^2^ was used to analyze the differential expression of NAP1L1 in HCC cancer based on The Cancer Genome Atlas (TCGA) database.

### Cell Culture

Normal liver cell lines (LO2) and HCC cell lines (HCCLM3, PLC/PRF-5, Huh-7, 97H, Hep-G2, and Hep-3B) were purchased from the Cell Bank of the Chinese Academy of Sciences (Shanghai, China) and cultured in Dulbecco’s modified Eagle medium (DMEM) (HyClone, Logan, UT, United States) supplemented with 10% fetal bovine serum (FBS; PAN-Biotech, Aidenbach, Germany). The cell lines were incubated with a 5% CO_2_ humidified chamber at 37°C.

### RT-PCR and QPCR

Total RNA was isolated from the HCC cell lines using a TRIzol Kit (Foregene, Chengdu, China). cDNA was synthesized using a cDNA synthesis kit (Vazyme, Nanjing, China), and the cDNA was used as a template for amplification using specific primers (GAPDH genes were used as internal gene controls). The primers used in this study are shown in Supplementary Table 1. RT-PCR and quantitative polymerase chain reaction (qPCR) were performed following the manufacturer’s instructions using Bio-Rad T100 and Bio-Rad CFX96 detection systems.

### Immunohistochemistry

This study was approved by the Research Ethics Committee of The Affiliated Hospital of Guizhou Medical University (No. 2018005). In this study, a total of 10 HCC tissues and 10 adjacent tissues were obtained from HCC patients undergoing surgical treatment.

Tissue array was purchased from Shanghai Tufei Biotech (Shanghai Tufeibio, Shanghai, China). It was used to examine NAP1L1 protein expression. The indirect streptavidin–peroxidase method was used following the manufacturer’s standard experiment guidelines. Cell staining was scored separately by two pathologists blinded to the clinical parameters. The extent of staining, defined as the percentage of positively stained tumor cells with respect to the whole tissue area, was scored on a scale of 0–4 as follows: 0, <10%; 1, 10–25%; 2, 26–50%; 3, 50–75%; and 4, >75%. The staining intensity was scored as 0–3 (Negative: 0; Weak expression: 1; Positive expression: 2; Strong expression: 3). The score represents the product of positive staining score, and the color intensity score was used as the final staining score for NAP1L1 (Abcam, Cambridge, MA, United States), Ki-67 (Cell Signaling Technology, Danvers, MA, United States), and PCNA (Proteintech, Wuhan, China) (0–12). For statistical analysis, final staining scores of 0–6 and 8–12 were considered to show low and high expressions, respectively. The Cat numbers, origins, and dilution concentrations used for all antibodies are listed in Supplementary Table 2.

### Lentivirus Infection

ShRNA-NAP1L1 lentiviral particles were constructed by GeneChem (Shanghai, China). HCCLM3 and Huh-7 cells were infected with the lentiviral vector. Silencing efficiency for NAP1L1 was measured by Western blot analysis. Transient and stable disturbance sequences are shown in Supplementary Table 3.

### siRNA and Plasmid Transfection

SiRNAs for NAP1L1 were designed and synthesized by RiboBio (Guangzhou, China). Plasmids for HDGF were purchased from Vigene Biosciences. Twelve hours before transfection, the HCC cells were plated into six-well plates (Nest Biotech, Wuxi, China) and cultured to 30–50% confluence. SiRNAs or plasmids were then transfected at a working concentration of 100 nM using the Lipofectamine 2000 Transfection Reagent (Invitrogen, Carlsbad, CA, United States) following the manufacturer’s protocol. Then, DMEM was supplemented with 10% FBS after 4 h.

### MTT Cytotoxicity

The HCC cancer cells (5,000/well) were seeded into 96-well plates. For lentivirus-mediated shNAP1L1 expression, the cells were incubated for a week. For transient transfections with si-NAP1L1, the cells were cultured for up to 4 days. Subsequently, 20 μl of MTT (5 μg/μl in PBS) (Sigma, St. Louis, MO, United States) solution was added to each well and incubated for 4 h. Then, the formazan crystals formed by viable cells were solubilized in 150 ml dimethyl sulfoxide (Sigma, St. Louis, MO, United States), and the absorbance value (OD) was measured at 490 nm. All the experiments were repeated at least three times.

### Colony Formation Assay

Cloning is based on the previous study (Lin et al., 2019). The cells were seeded in six-well culture plates at 200–500 cells/well (the number of inoculations was determined following the minimum of population dependence of cell lines). After incubation for 14 days, the cells were washed twice with PBS solution and stained with hematoxylin solution. The number of colonies was counted under a microscope. All the experiments were repeated at least three times.

### EdU Staining

In the EdU incorporation assay, the proliferating HCC cells were examined using a Cell-Light EdU Apollo 488 or 567 *In Vitro* Imaging Kit (RiboBio) following the manufacturer’s protocol. After incubation with 10 mM EdU for 2 hours, the HCC cells were treated with 4% paraformaldehyde, permeabilized in 0.3% Triton X-100, and stained with Apollo fluorescent dyes. A total of 5 mg/ml of DAPI was used to stain the cell nuclei for 10 min. The number of EdU-positive cells was counted under a fluorescence microscope in five random fields. All the assays were independently performed three times.

### Subcutaneous Tumorigenesis in Nude Mice

A total of 5 × 10^6^ logarithmically growing HCC cells of NAP1L1 downregulated and their corresponding negative control (NC) cells were injected into the subcutaneous tissues of nude mice (BALB/C, nu/nu, female 3–4 weeks old) (one group = 5). The animals were fed an autoclaved laboratory rodent diet. On the 21st day, the tumor tissues were excised and weighed. All the animal studies were conducted in accordance with the principles and procedures outlined in the Guizhou Medical University Guide for the Care and Use of Animals.

### Western Blot Assays

The extracted proteins were separated by 10% SDS-PAGE and further transferred onto PVDF membranes (Millipore, Bedford, MA, United States). Antibodies including NAP1L1 (Abcam), CCND1 (Abcam), HDGF (Proteintech), and c-Jun (Proteintech) were used in the Western blot assays following the manufacturer’s instructions. Detection was performed using ECL Plus Western blotting detection reagents (Millipore, United States). The specific protein expression levels of the blots were normalized to GAPDH (Santa). The Cat numbers, origins, and dilution concentrations used for all antibodies are listed in Supplementary Table 2.

### Co-Immunoprecipitation (Co-IP) Assay

Co-Immunoprecipitation (Co-IP) assay was carried out using Pierce Co-IP Kit (Thermo Scientific, United States) following the manufacturer’s instructions. The total protein was extracted and quantified. A total of 3,000 μg protein in 400 μl supernatant was incubated with 10 μg anti-NAP1L1 (Abcam), anti-HDGF (Proteintech), anti-c-Jun (Proteintech), or anti-IgG antibodies for 12 h at 4°C. The beads were washed, eluted in a sample buffer, and boiled for 10 min at 100°C. The immune complexes were subjected to Western blot analysis. Anti-IgG was used as an NC. The Cat numbers, origins, and dilution concentrations used for all antibodies are listed in Supplementary Table 2.

### Confocal Microscopy

HCCLM3 and Huh-7 cells were cultured overnight (2 × 10^5^/immunofluorescence well) and then treated with 4% paraformaldehyde and permeabilized with 0.5% Triton X-100 at room temperature. The cells were incubated with anti-NAP1L1 (Abcam), anti-HDGF (Proteintech), and anti-c-Jun (Proteintech) antibodies for 60 min at 37°C. After incubation for 30 min at 37°C with a secondary antibody, coverslips were mounted onto the slides with a mounting solution containing 0.2 mg/ml DAPI. The images were captured by laser scanning confocal microscopy (Zeiss LSM 800). The Cat numbers, origins, and dilution concentrations used for all antibodies are listed in Supplementary Table 3.

### Statistical Analysis

Statistical analyses were carried out using SPSS 22.0 statistical software package (SPSS, Chicago, IL, United States). The data are shown as the mean ± SD obtained from at least three independent experiments. Two-tailed Student’s *t*-test was applied for comparisons between groups. Survival analysis was performed using the Kaplan–Meier method. All statistical tests were two-sided; single, double, and triple asterisks indicate statistical significance (^∗^*p* < 0.05, ^∗∗^*p* < 0.01, and ^∗∗∗^*p* < 0.001).

## Results

### NAP1L1 Level Is Upregulated in HCC and Correlates With Poor Prognosis

**FIGURE 1:**
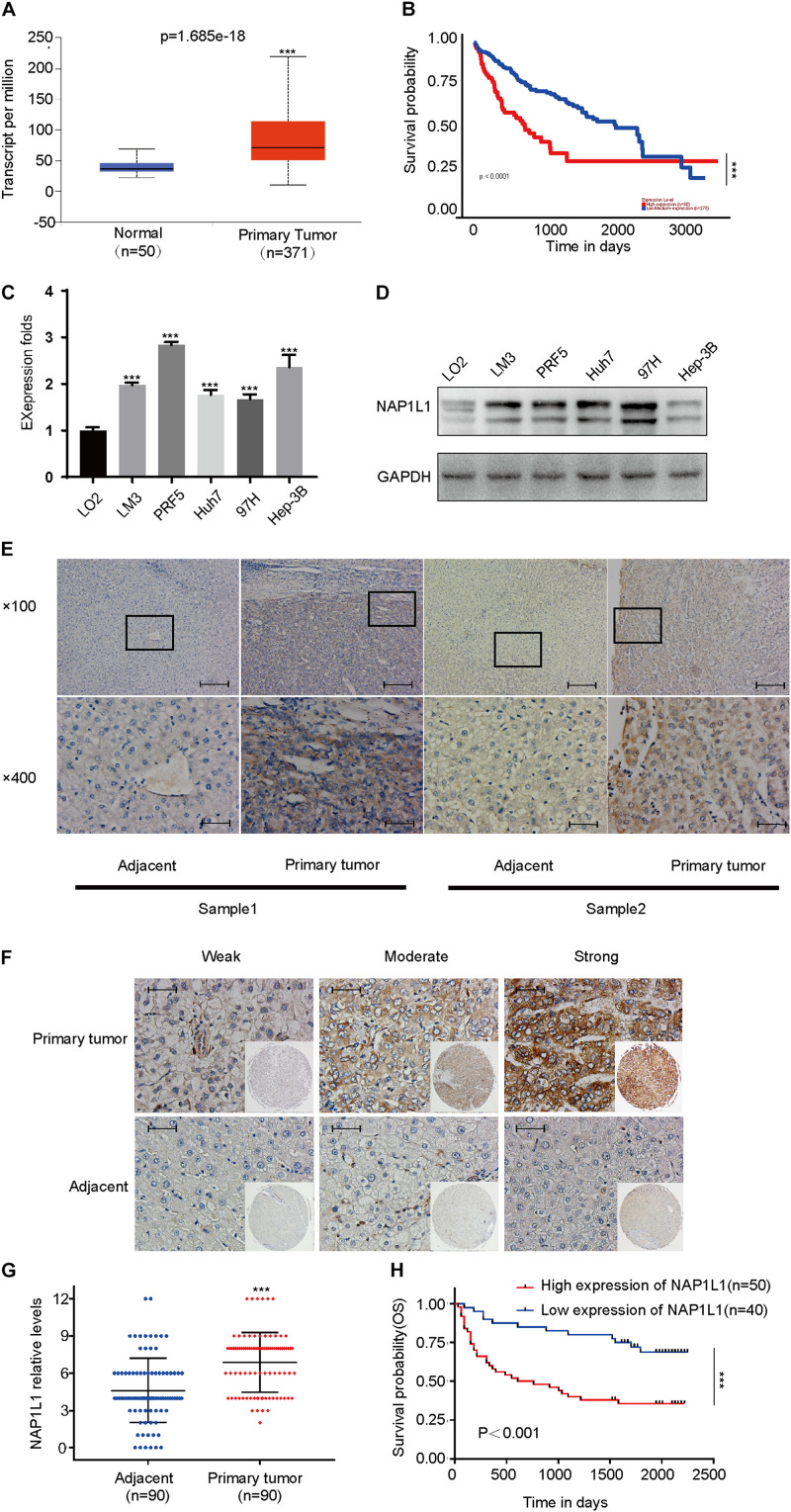
NAP1L1 was upregulated in HCC, and high expression reduced the overall survival. **(A)** NAP1L1 mRNA expression in liver cancer tissue and peritumoral tissue among the HCC patients obtained from the TCGA database (http://ualcan.path.uab.edu/). **(B)** Kaplan–Meier survival analysis for overall survival based on the NAP1L1 expression data. **(C)** RT-qPCR analysis of NAP1L1 mRNA expression in normal hepatocytes and HCC cell lines. **(D)** Western blot analysis of expression levels in normal hepatocytes and HCC cell lines. **(E)** NAP1L1 expression was measured *via* immunohistochemical staining in HCC and paracarcinoma tissues (×100 visual field, scale bar: 200 μm, ×400, scale bar: 50 μm). **(F)** NAP1L1 expression performed in TMA (scale bar: 50 μm). **(G)** TMA immunohistochemical cell staining score. **(H)** Kaplan–Meier survival analysis for overall survival in TMA showing NAP1L1 expression. Data are presented as mean ± SD from three independent experiments. *p < 0.05 vs. control; **p < 0.01; ***p < 0.001.

According to the analysis of the TCGA database, NAP1L1 mRNA was upregulated and predicted poor prognosis (Figures 1A,B). Subsequently, consistent with the prediction, the upregulated expression of NAP1L1 mRNA and protein levels was confirmed by real-time quantitative PCR (qRT-PCR), Western blot analysis of the human HCC and normal liver cell lines, and immunohistochemistry assay on the clinic human HCC tissue sections (Figures 1C–E). Immunohistochemical analysis of NAP1L1 was performed in tissue microarrays (TMA) containing 90 paired HCC samples and adjacent non-tumor tissues of human HCC, and cell staining was scored (Figures 1F,G). Survival analysis showed that overexpressed NAP1L1 is an unfavorable factor, reducing the overall survival time of HCC patients (Figure 1H), and then the clinical significance of NAP1L1 expression was assessed (Table 1). Features associated with the survival in univariate Cox regression analysis were as follows: clinical stage (*p* < 0.001), histological grade (*p* = 0.023), tumor scale (*p* = 0.008), recurrence (*p* = 0.035), tumor thrombus (*p* < 0.001), lymph metastasis (*p* = 0.009), AFP stage (*p* < 0.001), and NAP1L1 expression (*p* = 0.001). However, multivariate Cox regression analysis indicated that clinical stage (*p* = 0.007) was a predictor for poor survival compared with high NAP1L1 level (Table 2).

**TABLE 1:** Correlation of NAP1L1 expression with clinicopathological characteristics of patients with HCC.

Characteristics	*n*	NAP1L1 expression		
		High	Low	*p*
**Gender**				
Male	70	37(52.86%)	33(47.14%)	0.335
Female	20	13(65.00%)	7(35.00%)	
**Age(year)**				
≤50	40	24(60.00%)	16(40.00%)	0.448
>50	50	26(52.00%)	24(48.00%)	
**Clinical stage**				
I	58	25(43.10%)	33(56.90%)	0.001
II∼III	32	25(78.13%)	7(21.88%)	
**Tumor scale**				
≤5 cm	47	24(51.06%)	23(48.94%)	0.37
>5cm	43	26(60.47%)	17(39.53%)	
**Histological Grade**				
I∼II	54	22(40.74%)	32(59.26%)	<0.001
III	36	28(77.78%)	8(22.22%)	
**Vital states**				
Alive	46	18(39.13%)	28(60.87%)	0.001
Die	44	32(72.73%)	12(27.27%)	
**Recurrence**				
No	22	16(72.73%)	6(27.27%)	0.062
Yes	68	34(50.00%)	34(50.00%)	
**HBsAg**				
Positive	70	40(57.14%)	30(42.86%)	0.571
Negative	20	10(50.00%)	10(50.00%)	
**Tumor thrombus**				
No	65	29(44.62%)	36(55.38%)	<0.001
Yes	25	21(84.00%)	4(16.00%)	
**Lymph metastasis metastasismetastasis**				
No	86	48(55.81%)	38(44.19%)	0.819
Yes	4	2(50.00%)	2(50.00%)	
**AFP stage**				
≤200 μg/L	45	15(33.33%)	30(66.67%)	<0.001
>200 μg/L	45	35(77.78%)	10(22.22%)	

**TABLE 2:** Summary of univariate and multivariate Cox regression analysis.

Parameter	Univariate analysis			Multivariate analysis		
	Hazard Ratio	95.0% CI	*p*	Hazard Ratio	95.0% CI	*p*
Gender	1.448	0.745-2.814	0.275			
Age(year)	0.702	0.388-1.271	0.243			
Clinical stage	5.203	2.809-9.639	<0.001	2.97	1.349-6.536	0.007
Tumor scale	2.291	1.246-4.214	0.008	1.752	0.906-3.390	0.096
Histological grade	1.995	1.102-3.611	0.023	0.851	0.429-1.689	0.645
Recurrence	0.501	0.263-0.954	0.035	0.495	0.237-1.035	0.062
HBsAg	0.947	0.468-1.917	0.88			
Tumor thrombus	3.614	1.973-6.622	<0.001	1.375	0.671-2.819	0.385
Lymph metastasis	4.007	1.424-11.273	0.009	3.159	0.947-10.535	0.061
AFP stage	3.124	1.648-5.923	<0.001	1.764	0.803-3.871	0.157
NAP1L1 expression	3.135	1.609-6.110	0.001	2.016	0.871-4.667	0.102

### NAP1L1 Knockdown Inhibits Cell Proliferation *in vitro* and *in vivo*

**FIGURE 2:**
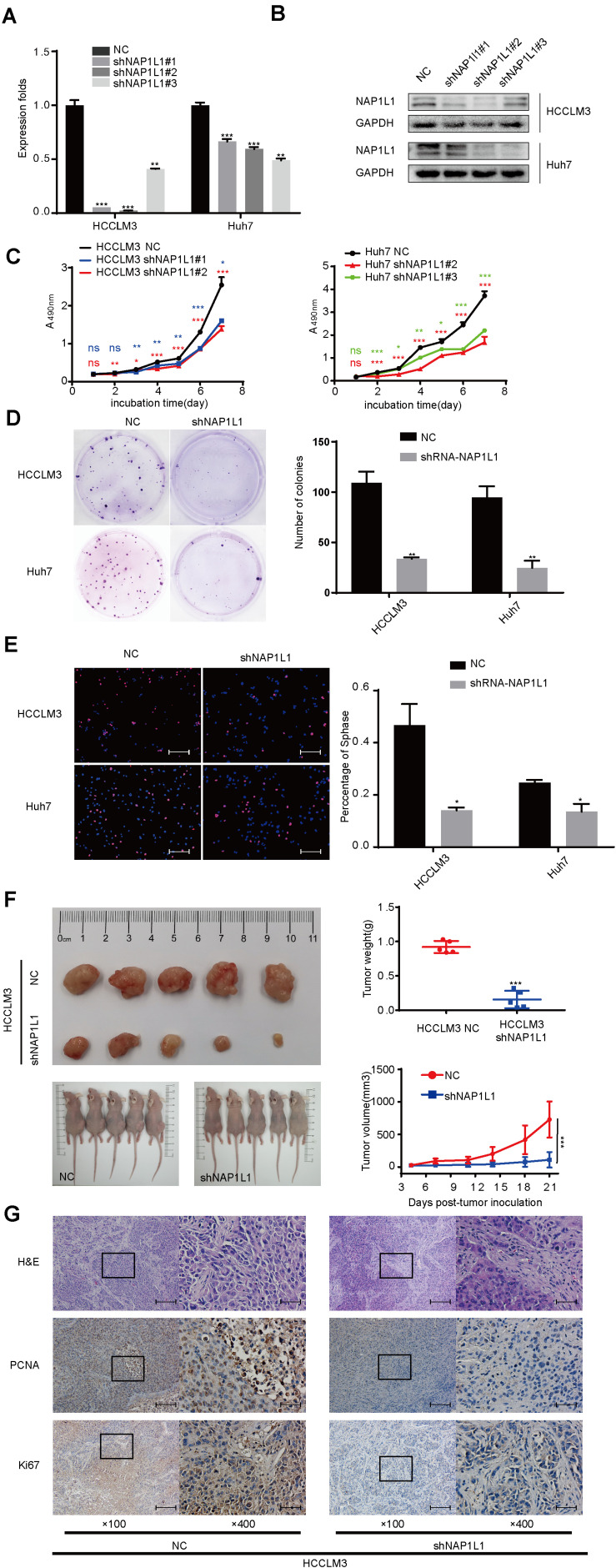
Downregulated NAP1L1 expression attenuated cell proliferation. **(A)** Quantitative RT-qPCR data to measure gene expression after shRNA-NAP1L1 lentivirus or NC lentivirus transfection into HCCLM3 and Huh7 cells. **(B)** Efficiency of silencing NAP1L1 by Western blot analysis. **(C)** MTT assays indicated that shRNA-NAP1L1 inhibited the proliferation Continued *in vitro*. **(D)** Downregulated NAP1L1 expression suppressed plate clone formation. **(E)** EdU assay showed that the downregulation of NAP1L1 suppressed the proliferation *in vitro* (scale bar: 200 μm). **(F)** Xenograft tumor of nude mice showed that the average weight and volume of tumors decreased in the shRNA-NAP1L1 group compared with the NC group. **(G)** Hematoxylin–eosin staining in xenograft tumor of nude mice and expression of PCNA and Ki-67 were measured *via* IHC staining in the xenograft tumor of nude mice (×100 visual field, scale bar: 200 μm, ×400, scale bar: 50 μm). Data are presented as mean ± SD from three independent experiments. *p < 0.05 vs. control; **p < 0.01; ***p < 0.001.

To investigate the NAP1L1 effects on HCC proliferation, lentivirus-carrying shRNA-NAP1L1 was infected into HCCLM3 and Huh7 cells (Supplementary Figure 1A). The transfection efficiency was first analyzed by a qRT-PCR analysis (Figure 2A), and Western blot analyses were used to further verify this result (Figure 2B). Subsequently, the MTT (Figure 2C), plate clone (Figure 2D), and EdU staining assays confirm that shNAP1L1 inhibits cell proliferation *in vitro* (Figure 2E). A preliminary *in vivo* study was carried out. The average weight and volume of tumors significantly decreased in those xenograft mice injected with NAP1L1-level-decreased HCC cells compared with the NC group (Figure 2F and Supplementary Figure 1B). Then, ki-67 and PCNA expressions in xenograft tumors in nude mice were detected, and it was confirmed that in the group injected with shRNA-NAP1L1, the content of tumors was significantly smaller than that of the NC xenograft group (Figure 2G).

### siRNA-NAP1L1 Reduces Cell Proliferation *in vitro*

**FIGURE 3:**
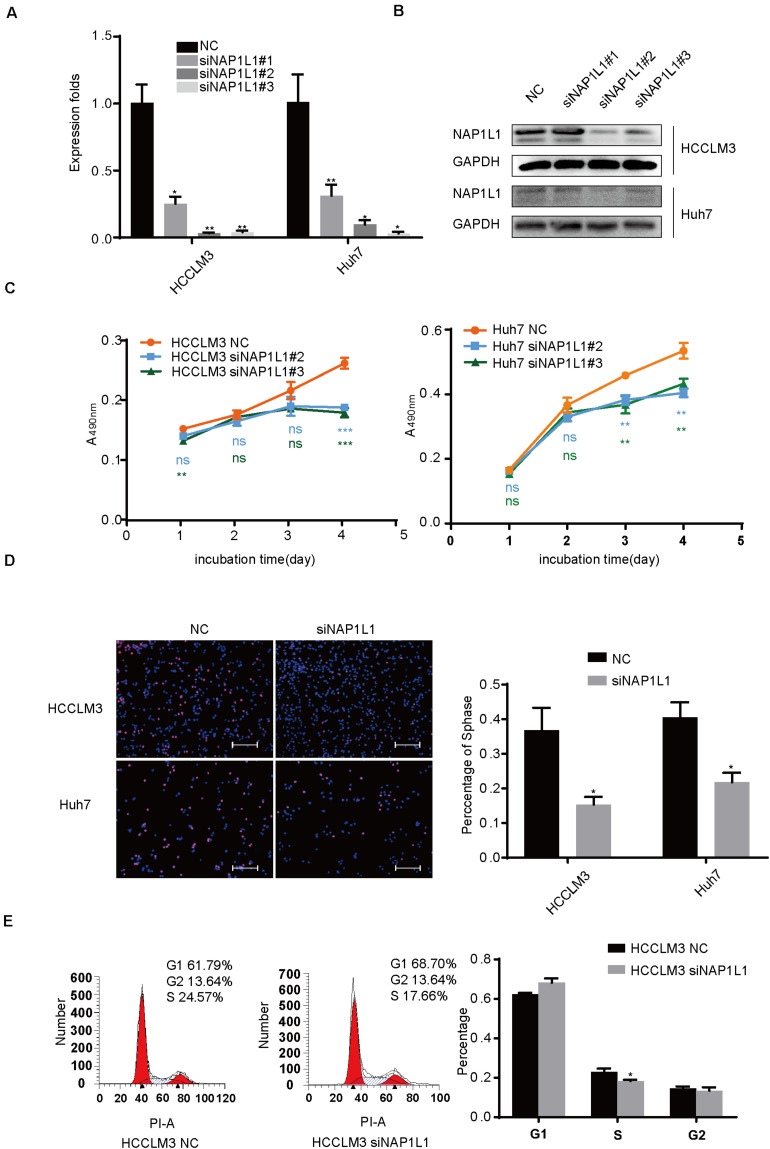
SiRNA-NAP1L1 reduces cell proliferation *in vitro.* **(A)** Quantitative RT-qPCR data to measure gene expression after siRNA-NAP1L1 or NC lentivirus transfection into HCCLM3 and Huh7 cells. **(B)** Expression levels of NAP1L1 were detected by Western blot analysis to screen effective transfection fragments. **(C)** MTT assays showed that the inhibition of NAP1L1 reduces proliferation *in vitro* in HCCLM3 and Huh7. **(D)** EdU assay indicated that the downregulation of NAP1L1 suppressed the proliferation *in vitro* (scale bar: 200 μm). **(E)** Cell cycle distribution was subjected to flow cytometry, and quantified histograms show the effect of NAP1L1 downregulation on cell cycle distribution. Data are presented as mean ± SD from three independent experiments. *p < 0.05 vs. control; **p < 0.01; ***p < 0.001.

The HCC cancer cells were transfected with siRNA-NAP1L1, and their knockdown efficiency was verified (Figures 3A,B). SiRNAs which significantly repressed the expression of NAP1L1 were selected to perform MTT and EdU assays. The MTT assay shows that the reduced NAP1L1 protein level significantly decreased the cell growth (Figure 3C). EdU staining assay confirms the results obtained in the MTT assay (Figure 3D). We also assessed the effect of NAP1L1 in downregulation on cell cycle distribution by flow cytometry. The results show that the knockdown of NAP1L1 inhibited the S phase of HCC cells *in vitro* (Figure 3E).

Moreover, we also assessed the effect of NAP1L1 overexpression on downstream signaling. This was associated with the increase in the expression of HDGF, c-Jun, and CCND1 (Supplementary Figures 1A,B). The MTT and EdU assay showed that the increased NAP1L1 protein level significantly promoted the cell growth (Supplementary Figures 1C,D). Overexpression of NAP1L1 promoted HCC proliferation by promoting G1/S transition (Supplementary Figure 1E).

### NAP1L1 Interacts With HDGF

**FIGURE 4:**
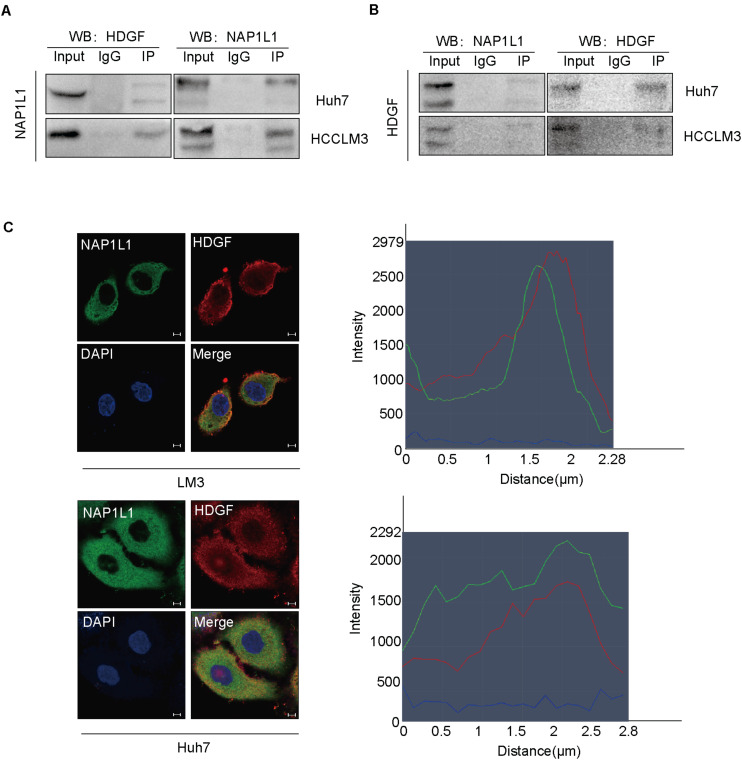
NAP1L1 interacts with HDGF. **(A,B)** Co-IP assay identified NAP1L1 coprecipitated with HDGF. **(C)** Confocal microscopic images showed the colocalization of NAP1L1 and HDGF in the cytoplasm in HCCLM3 and Huh7 cells (scale bar: 5 μm).

Interestingly, a previous study in our group had found that NAP1L1 is a potential candidate of HDGF interaction proteins in endometrial carcinoma using exogenous Co-IP combined with mass spectrometry (unpublished data). In this study, we confirmed that NAP1L1 binds to HDGF by an endogenous Co-IP assay (Figures 4A,B). Furthermore, the confocal microscopic images showed the colocalization of NAP1L1 and HDGF in the cytoplasm of HCC cells (Figure 4C).

### NAP1L1 Recruits c-Jun

**FIGURE 5:**
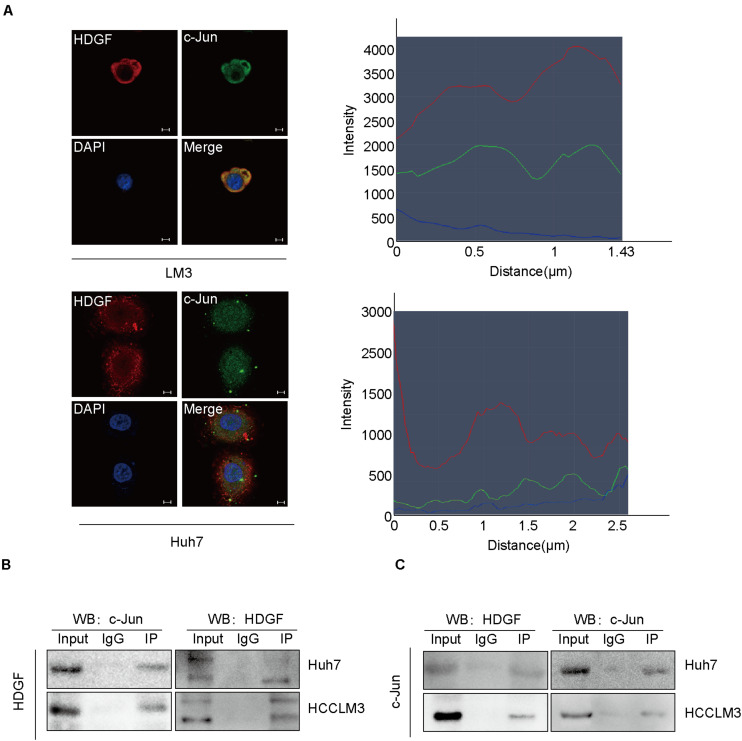
NAP1L1 interacts with HDGF to recruit c-Jun. **(A)** Confocal microscopic images showed the colocalization of HDGF and c-Jun in the cytoplasm and nucleus (predominantly in the cytoplasm) in HCCLM3 and Huh7 cells (scale bar: 5 μm). **(B,C)** Co-IP assay was performed to determine that HDGF co-precipitated with c-Jun.

Furthermore, we observed that HDGF interacted with c-Jun by Co-IP assay both in the cytoplasm and in the nucleus, predominantly in the cytoplasm (Figure 5A). c-Jun is an oncogenic transcription factor significantly participating in many tumor pathogeneses by transcription or suppressing the expression of some genes. Previous studies have shown that c-Jun regulates the proliferation of non-small cell lung cancer by targeting CCND1 (Zhao et al., 2018). The Co-IP assay in this study showed the interaction between c-Jun and HDGF in HCC (Figures 5B,C).

### Transfecting HDGF Increases c-Jun/CCND1 Signal and Restores Cell Proliferation in NAP1L1-Suppressing HCC Cells

**FIGURE 6:**
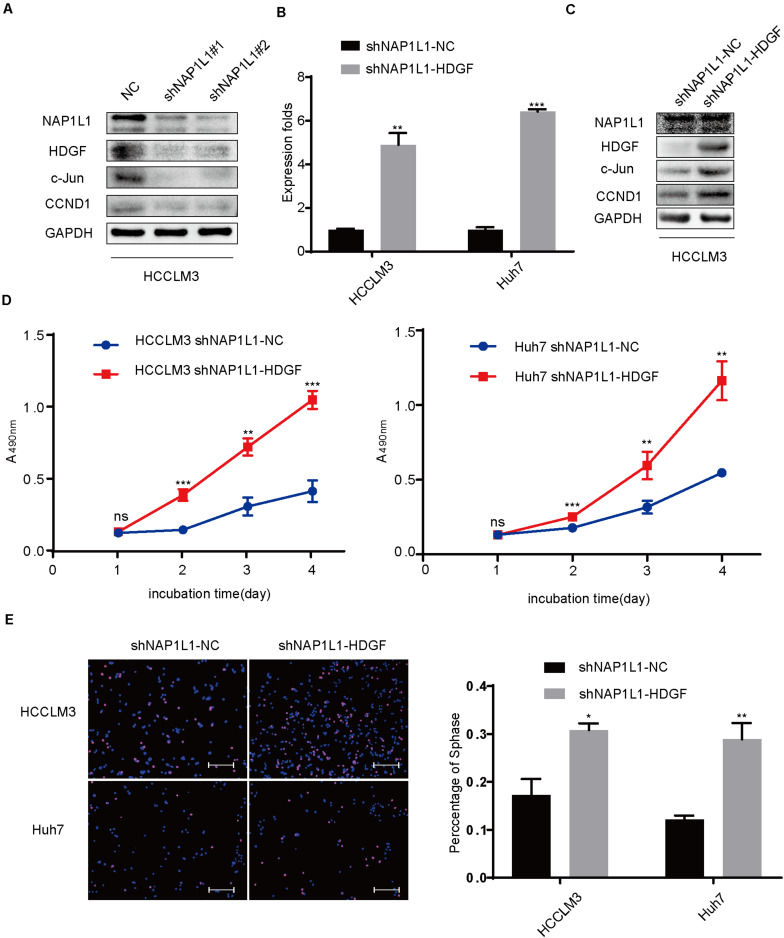
Transfecting HDGF restores cell proliferation in NAP1L1-suppressing HCC cells through the c-Jun/CCND1 signal. **(A)** NAP1L1, HDGF, c-Jun, and CCND1 protein expression in NC and shNAP1L1 cells in HCCLM3. **(B)** RT-qPCR data to measure HDGF expression after HDGF is restored in shNAP1L1 HCC cells. **(C)** NAP1L1, HDGF, c-Jun, and CCND1 protein sequences in HDGF restored shNAP1L1 HCCLM3. **(D)** MTT assays indicated that transfecting HDGF restores cell proliferation. **(E)** EdU assay indicated that transfecting HDGF restores cell proliferation (scale bar: 200 μm). Data are presented as mean ± SD from three independent experiments. *p < 0.05 vs. control; **p < 0.01; ***p < 0.001.

In this study, the NAP1L1 knockdown efficiencies and change in downstream were validated by Western blot assays (Figure 6A). The HDGF cDNA plasmid was transfected to NAP1L1-suppressing cells, to explore the role of HDGF in NAP1L1-mediated pathogenesis of HCC cancer. The knockdown efficiency was verified by qRT-PCR and western blot analysis (Figures 6B,C), and according to our observation *in vitro*, the ability of cell proliferation (Figure 6D) and EdU staining (Figure 6E) was restored. Western blot assay indicated that the c-Jun/CCND1 signal was significantly increased (Figure 6C).

### Transfecting c-Jun Increases CCND1 Signal and Restores Cell Proliferation in NAP1L1-Suppressing HCC Cells

**FIGURE 7:**
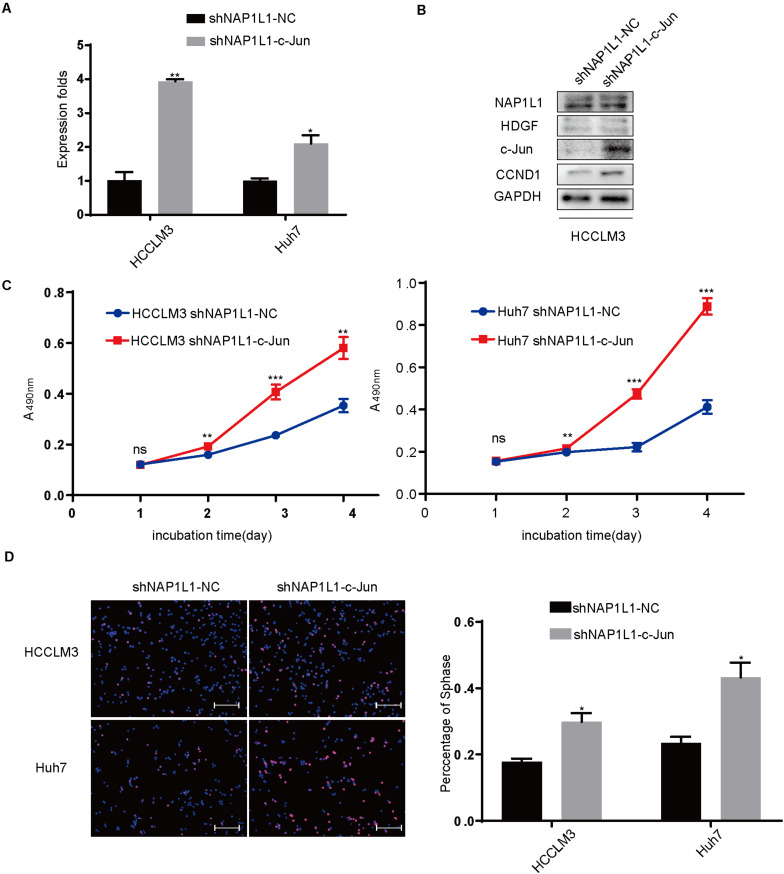
Transfecting c-Jun increases the CCND1 signal and restores cell proliferation in NAP1L1-suppressing HCC cells. **(A)** Quantitative RT-qPCR data to measure c-Jun expression after restoring c-Jun in shNAP1L1 HCC cells. **(B)** NAP1L1, HDGF, c-Jun, and CCND1 protein sequences in HDGF restored shNAP1L1 HCCLM3 (scale bar: 200 μm). **(C)** MTT assays showed that transfecting c-Jun restores cell proliferation. **(D)** EdU assay showed that transfecting c-Jun restores cell proliferation (scale bar: 200 μm). *p < 0.05 vs. control; **p < 0.01; ***p < 0.001.

In addition, we transfected the c-Jun cDNA plasmid to NAP1L1-suppressing cells. qRT-PCR and Western blot analysis (Figures 7A,B) verified the knockdown efficiency, and the ability of cell proliferation (Figure 7C) and EdU staining (Figure 7D) was restored *in vitro*.

## Discussion

Hepatocellular carcinoma accounts for most liver cancers. This type of cancer occurs more often in men than in women. It is usually diagnosed in people of age 50 or older. In China, HCC is one of the common malignant tumors and mostly correlated with chronic HBV infection and subsequent liver cirrhosis formation. In the pathogenesis of HCC, a large number of genes and signal pathways show abnormal expression (Chen et al., 2019; Luiken et al., 2020). Therefore, exploring the pathogenesis of HCC will help to further improve the treatment of HCC, improving the survival prognosis and prolonging the survival time of patients.

The human NAP1-like protein (NAP1L) family comprises NAP1L1, NAP1L2, NAP1L3, NAP1L4, NAP1L5, and NAP1L6 proteins (Attia et al., 2013). In recent studies, NAP1L1 has been shown as a promoter of tumor pathogenesis. NAP1L1 participates in the miR-532-5p-mediated suppression of renal cancer cell proliferation. In colorectal cancer and pancreatic neuroendocrine neoplasm, NAP1L1 was found to be a biomarker, involved in the pathogenesis of these two types of cancers (Schimmack et al., 2014; Zhai et al., 2018; Queiroz et al., 2020). In HCC, NAP1L1 acts as a tumor promoter and is repressed by PRDM8 and let-7c-5p. However, the molecular basis of NAP1L1 in modulating HCC proliferation is still undermined.

In this study, we first analyzed the expression of NAP1L1 mRNA in the TCGA database. The data showed that the NAP1L1 expression level increased in HCC. Furthermore, the higher the malignant grade of HCC, the higher the expression level of NAP1L1. Survival analysis showed that a high expression of NAP1L1 significantly shortened the overall survival time of HCC patients. These data indicate that NAP1L1 is a potentially significant oncogene in HCC. To confirm these data, qPCR was used to examine the NAP1L1 mRNA expression in HCC tissues and cells compared with liver tissues and cells. The results are consistent with the TCGA data of NAP1L1. Furthermore, immunohistochemistry was used to test the NAP1L1 protein expression. Consistent with the mRNA data, the expression of NAP1L1 was found to be higher in HCC cell lines and tissues. Our results are similar to other reports of NAP1L1 in HCC cancer, indicating that NAP1L1 is a tumor promoter participating in HCC pathogenesis.

In previous studies, NAP1L1 has been reported to be involved in promoting tumor pathogenesis (Schimmack et al., 2014; Zhai et al., 2018; Queiroz et al., 2020), but few studies have shown its role in cancers. Here, the role and molecular basis of NAP1L1 in HCC were further explored. It was observed that the suppression of NAP1L1 by siRNA or shRNA significantly decreased the cell cycle transition and cell proliferation *in vivo* and *in vitro* by MTT assay, plate clone formation, EdU staining, and subcutaneous tumorigenesis in nude mice. These data further support that NAP1L1 is a potential oncogene in HCC.

Previous studies showed that NAP1L1 is repressed by PRDM8 and let-7c-5p (Chen et al., 2018; Huang et al., 2018) involved in HCC pathogenesis. Here, we found a new molecular basis for NAP1L1 to modulate HCC growth. HDGF was primarily obtained from the conditioned media of Huh-7 hepatoma cells (Nakamura et al., 1989). HDGF has been widely documented as an oncogene, inducing tumor occurrence and development including endometrial cancer, nasopharyngeal carcinoma, non-small cell lung cancer, liver cancer, and breast cancer (Wang and Fang, 2011; Chen et al., 2012; Fu et al., 2017; Min et al., 2018; Liu C. et al., 2019; Xiao et al., 2019).

Use of HDGF antibody therapy has significantly increased the antineoplastic activity of gemcitabine, bevacizumab, and chemotherapy in non-small cell lung cancer (Ren et al., 2009; Zhao et al., 2013). These studies show the significance of HDGF in the occurrence of malignant tumors.

Interestingly, previous studies in our lab had found that NAP1L1 is a potential candidate of HDGF interaction proteins in endometrial carcinoma using exogenous Co-IP assay combined with mass spectrometry (unpublished data). Furthermore, endogenous Co-IP assay and microconfocal colocalization assay were used to determine that NAP1L1 interacted with HDGF and colocalized in the cytoplasm and nucleus. To further elucidate the molecular mechanism of NAP1L1 for promoting cell cycle transition and cell proliferation *via* HDGF, the BioGrid database was used to predict the interacting proteins of HDGF and observed that c-Jun is a potential candidate. c-Jun is an oncogenic transcription factor, significantly participating in many tumor pathogeneses by transcription or suppressing the expression of some genes (Lin et al., 2019, 2020; Liu Y. et al., 2019; Liu et al., 2020; Zou et al., 2020). In HCC, c-Jun has been reported to modulate cell cycle signals, promoting malignant phenotypes of HCC (Eferl et al., 2003; Machida et al., 2010; Min et al., 2012), indicating the key role of c-Jun in HCC. Then, by consulting the BioGrid database, a combination was found between Jun and HDGF (Wang et al., 2011). In subsequent study, endogenous Co-IP assay showed that HDGF binds to c-Jun. Furthermore, HDGF and c-Jun were shown to be colocalized in the cytoplasm and nucleus. The results indicate that HDGF recruits c-Jun to participate in HCC carcinogenesis.

Finally, HDGF or c-Jun cDNA plasmid was transfected into shNAP1L1-treated HCC cells, and it was found that the cell cycle transition signal was significantly increased in shNAP1L1-treated HCC cells. Furthermore, the cell proliferation ability was also restored in NAP1L1-suppressed HCC cells. These data revealed that HDGF or c-Jun positively participated in NAP1L1-induced HCC proliferation.

In summary, elevated NAP1L1 protein level is a significantly unfavorable outcome for HCC patients. It acts as a tumor promoter that binds to HDGF. The latter recruits c-Jun to stimulate cell cycle signal transition and thus induces HCC carcinogenesis.

However, the results reported in this study are not sufficient to determine whether NAP1L1 interacts with HDGF and c-Jun to form a protein complex, or interacts with HDGF, further affecting the interaction between HDGF and c-Jun. This will be studied further in the future.

## Data Availability Statement

The datasets presented in this study can be found in online repositories. The names of the repository/repositories and accession number(s) can be found in the article/Supplementary Material.

## Ethics Statement

The studies involving human participants were reviewed and approved by the Research Ethics Committee of The Affiliated Hospital of Guizhou Medical University. The patients/participants provided their written informed consent to participate in this study. The animal study was reviewed and approved by the Research Ethics Committee of The Affiliated Hospital of Guizhou Medical University. Written informed consent was obtained from the individual(s) for the publication of any potentially identifiable images or data included in this article.

## Author Contributions

SZ and SL planned the experiments and revised the manuscript. Y-wZ, QC, and BL performed the experiments and prepared a draft of the manuscript. H-YL and X-KZ performed the statistical analysis. Y-wZ conceived the project and edited the manuscript. Y-yX and Y-wZ discussed the results. All the authors read and approved the final manuscript.

## Conflict of Interest

The authors declare that the research was conducted in the absence of any commercial or financial relationships that could be construed as a potential conflict of interest.

## Publisher’s Note

All claims expressed in this article are solely those of the authors and do not necessarily represent those of their affiliated organizations, or those of the publisher, the editors and the reviewers. Any product that may be evaluated in this article, or claim that may be made by its manufacturer, is not guaranteed or endorsed by the publisher.

## Footnotes

1 https://thebiogrid.org/

2 http://ualcan.path.uab.EdU/

## Supplementary Material

Supplementary Figure 1 **(A)** Quantitative RT-qPCR data to measure the gene expression after NAP1L1 overexpression plasmid transfection into HCCLM3 and Huh7 cells. **(B)** Expression levels of NAP1L1 were detected by Western blot analysis to screen effective transfection fragments. **(C)** MTT assays showed that the upregulation of NAP1L1 promoted the proliferation *in vitro* in HCCLM3 and Huh7. **(D)** EdU assay indicated that the upregulation of NAP1L1 promoted the proliferation *in vitro* (scale bar: 200 μm). **(E)** Cell cycle distribution was subjected to flow cytometry, and quantified histograms show the effect of NAP1L1 overexpression on cell cycle distribution. Data are presented as mean ± SD from three independent experiments. ^∗^*P* < 0.05 vs. control; ^∗∗^*P* < 0.01; ^∗∗∗^*P* < 0.001.

Supplementary Figure 2 **(A)** Representative images of HCC cells stably transfected with fluorescently labeled lentivirus under bright-field and fluorescence microscopy (scale bar: 250 μm). Both HCCLM3 and Huh7 cells were transfected with NC lentivirus or shNAP1L1 lentivirus. **(B)** Xenograft tumor in nude mice in shRNA-NAP1L1 group compared with NC group.

Supplementary Figure 3 **(A)** EdU assay for the downregulation of NAP1L1 with shRNA-NAP1L1 lentivirus *in vitro* (scale bar: 200 μm). **(B)** EdU assay for the downregulation of NAP1L1 with siRNA-NAP1L1 *in vitro* (scale bar: 200 μm). **(C)** EdU assay indicated that transfecting HDGF restores the cell proliferation *in vitro* (scale bar: 200 μm). **(D)** EdU assay showed that transfecting c-Jun restores the cell proliferation *in vitro* (scale bar: 200 μm). **(E)** EdU assay indicated that the upregulation of NAP1L1 promoted the proliferation *in vitro* (scale bar: 200 μm).
