# Supplementary figures and images for: NAP1L1 Functions as a Tumor Promoter via Recruiting Hepatoma-Derived Growth Factor/c-Jun Signal in Hepatocellular Carcinoma
Source: Front Cell Dev Biol. 2021 Jul 23;9:659680. doi: 10.3389/fcell.2021.659680 (PMC8343235; doi:10.3389/fcell.2021.659680)

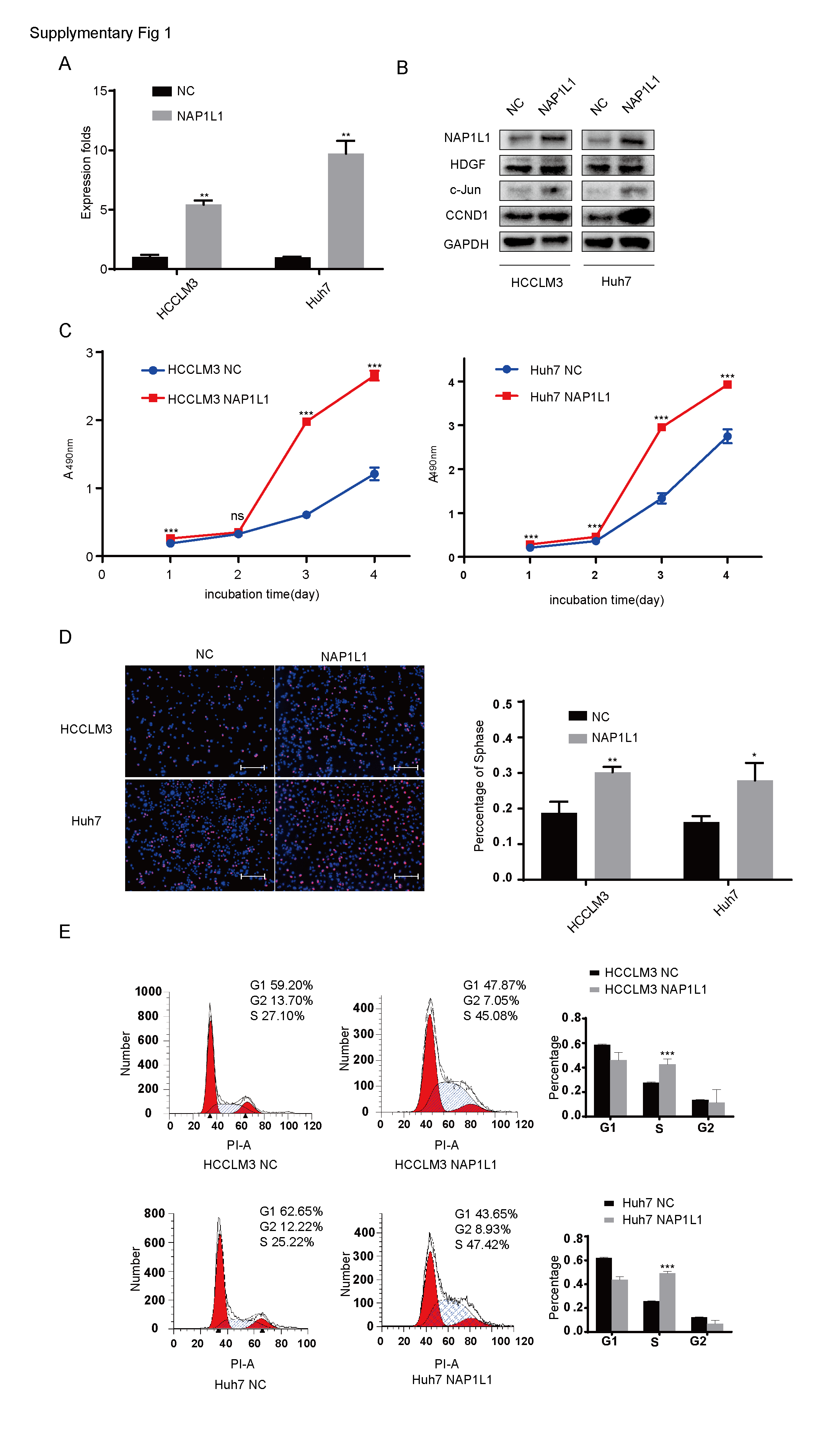

Supplement: Supplementary Figure 1 — (A) Quantitative RT-qPCR data to measure the gene expression after NAP1L1 overexpression plasmid transfection into HCCLM3 and Huh7 cells. (B) Expression levels of NAP1L1 were detected by Western blot analysis to screen effective transfection fragments. (C) MTT assays showed that the upregulation of NAP1L1 promoted the proliferation in vitro in HCCLM3 and Huh7. (D) EdU assay indicated that the upregulation of NAP1L1 promoted the proliferation in vitro (scale bar: 200 μm). (E) Cell cycle distribution was subjected to flow cytometry, and quantified histograms show the effect of NAP1L1 overexpression on cell cycle distribution. Data are presented as mean ± SD from three independent experiments. ∗P < 0.05 vs. control; ∗∗P < 0.01; ∗∗∗P < 0.001. [file Image_1.TIF]

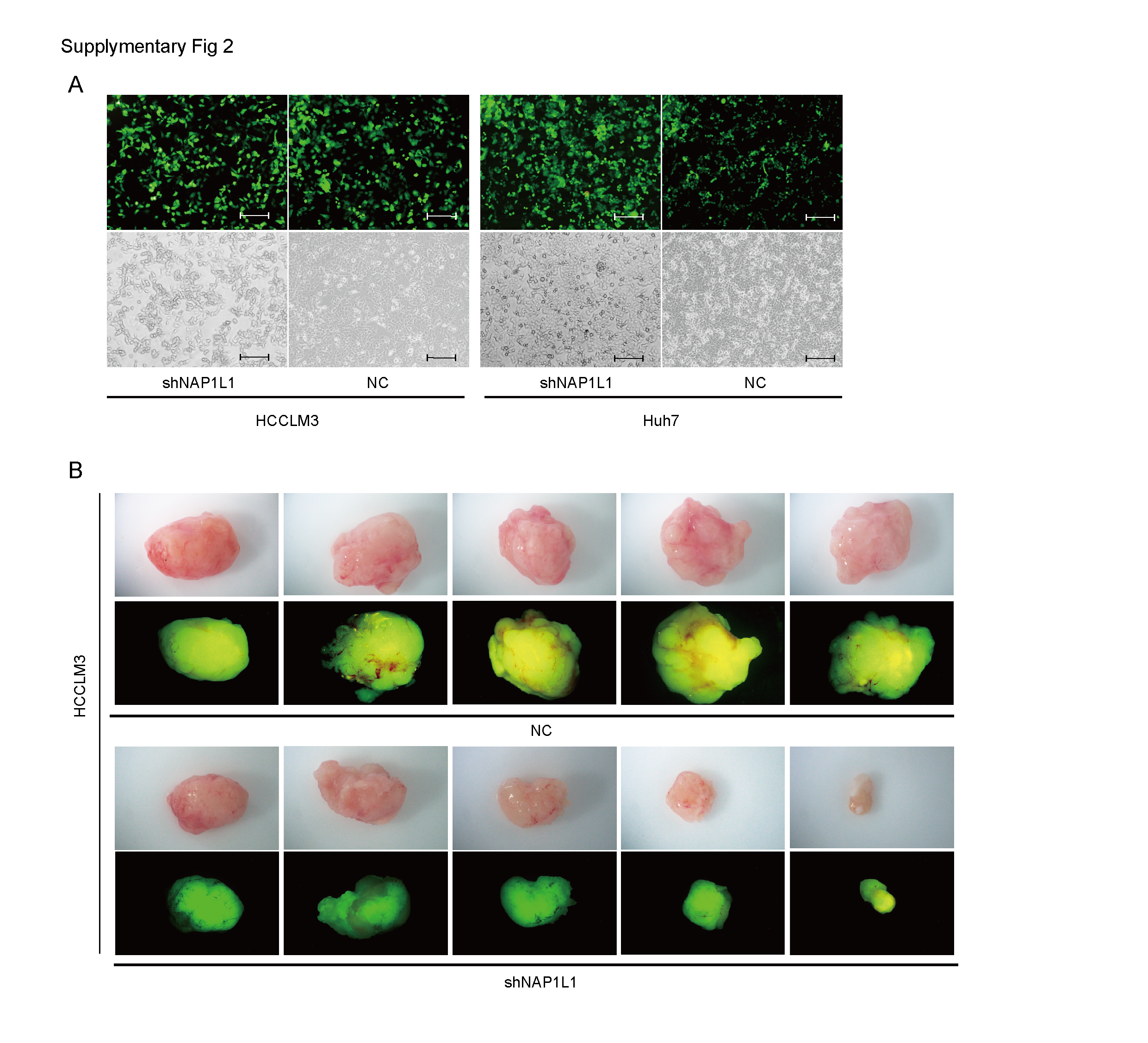

Supplement: Supplementary Figure 2 — (A) Representative images of HCC cells stably transfected with fluorescently labeled lentivirus under bright-field and fluorescence microscopy (scale bar: 250 μm). Both HCCLM3 and Huh7 cells were transfected with NC lentivirus or shNAP1L1 lentivirus. (B) Xenograft tumor in nude mice in shRNA-NAP1L1 group compared with NC group. [file Image_2.TIF]

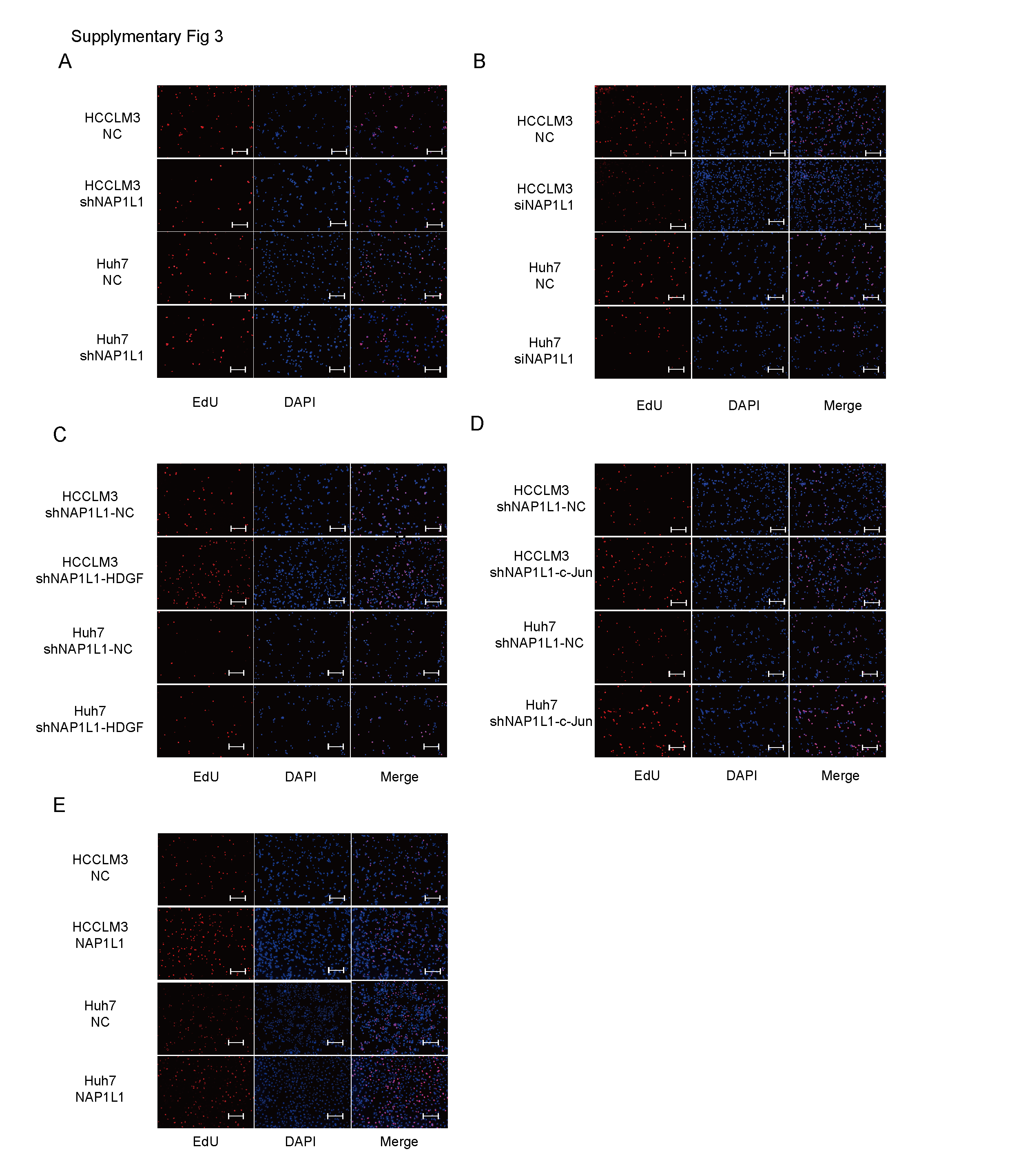

Supplement: Supplementary Figure 3 — (A) EdU assay for the downregulation of NAP1L1 with shRNA-NAP1L1 lentivirus in vitro (scale bar: 200 μm). (B) EdU assay for the downregulation of NAP1L1 with siRNA-NAP1L1 in vitro (scale bar: 200 μm). (C) EdU assay indicated that transfecting HDGF restores the cell proliferation in vitro (scale bar: 200 μm). (D) EdU assay showed that transfecting c-Jun restores the cell proliferation in vitro (scale bar: 200 μm). (E) EdU assay indicated that the upregulation of NAP1L1 promoted the proliferation in vitro (scale bar: 200 μm). [file Image_3.TIF]
