# Supplementary material for: NAP1L1 Functions as a Tumor Promoter via Recruiting Hepatoma-Derived Growth Factor/c-Jun Signal in Hepatocellular Carcinoma
Source: Front Cell Dev Biol. 2021 Jul 23;9:659680. doi: 10.3389/fcell.2021.659680 (PMC8343235; doi:10.3389/fcell.2021.659680)
Supplement: Supplementary file 4 [file Table_1.doc]

| Table S1.The primers used in this study | | |
| --- | --- | --- |
| Primers name |  | Sequence(5’-3’) |
| NAP1L1 | Forward | TTTGCCCCTCCTGAAGTTCC |
|  | Reverse | CCCAACACAACTTGAGACATCC |
| HDGF | Forward | ATCAACAGCCAACAAATACC |
|  | Reverse | TTCTTATCACCGTCACCCT |
| β-actin | Forward | ACAGAGCCTCGCCTTTGCC |
|  | Reverse | GATATCATCATCCATGGTGAGCTGG |
| c-JUN | Forward | TCAGACAGTGCCCGAGATG |
|  | Reverse | CTGCTGCGTTAGCATGAGTT |
| GAPDH | Forward | CATGGGTGTGAACCATGAGA |
|  | Reverse | GTCTTCTGGGTGGCAGTGAT |
