# Supplementary material for: NAP1L1 Functions as a Tumor Promoter via Recruiting Hepatoma-Derived Growth Factor/c-Jun Signal in Hepatocellular Carcinoma
Source: Front Cell Dev Biol. 2021 Jul 23;9:659680. doi: 10.3389/fcell.2021.659680 (PMC8343235; doi:10.3389/fcell.2021.659680)
Supplement: Supplementary file 5 [file Table_2.doc]

| Table S2.A list of Antibodies used for WB,Co-IP,IF and IHC. | | | | |
| --- | --- | --- | --- | --- |
| antibodies | Cat.No | Company | Species | Dulution |
| NAP1L1 | mAb ab178687 | Abcam | Rabbit | 1:10000(WB);1:200(IF);  1:20(Co-IP) |
| NAP1L1 | pAb#14898-1-AP | Proteintech | Rabbit | 1:1000(WB) |
| HDGF | mAb#60064-1-Ig | Proteintech | Mouse | 1:1000(WB);1:50(IF);  1:10(Co-IP) |
| HDGF | pAb 11344-1-AP | Proteintech | Rabbit | 1:1000(WB) |
| c-JUN | mAb#9165 | Cell Sigaling | Rabbit | 1:1000(WB);1:50(IF);  1:10(Co-IP) |
| CCND1 | mAb#6086-1-Ig | Proteintech | Mouse | 1:1000(WB) |
| PCNA | mAb#13110 | Cell Sigaling | Rabbit | 1:400(IHC) |
| Ki-67 | mAb#9449 | Cell Sigaling | Mouse | 1:400(IHC) |
| GAPDH | pAb AP0063 | Bioworld | Rabbit | 1:10000(WB) |
