# Supplementary material for: NAP1L1 Functions as a Tumor Promoter via Recruiting Hepatoma-Derived Growth Factor/c-Jun Signal in Hepatocellular Carcinoma
Source: Front Cell Dev Biol. 2021 Jul 23;9:659680. doi: 10.3389/fcell.2021.659680 (PMC8343235; doi:10.3389/fcell.2021.659680)
Supplement: Supplementary file 6 [file Table_3.doc]

| Table S3.Transient and stable disturbance sequences | | | |
| --- | --- | --- | --- |
| Gene | **Accession** | **NO.** | **Target Seq** |
| NAP1L1 | stB0007176A | st-h-NAP1L1_001 | GAAGTATGCTGTTCTCTAT |
|  | stB0007176B | st-h-NAP1L1_002 | CCAACAGGATACATTGAAA |
|  | stB0007176C | st-h-NAP1L1_003 | GACAGTTCGTACTGTGACT |
| NAP1L1 | NM_004537 | NAP1L1-RNAi(78814-1) | GCCAAGATTGAAGATGAGAAA |
|  | NM_004537 | NAP1L1-RNAi(78815-1) | TTGATAAGCGATTTGAAATTA |
|  | NM_004537 | NAP1L1-RNAi(78816-1) | TTCCAATGACTCTTTCTTTAA |
