# Supplementary material for: NAP1L1 Functions as a Tumor Promoter via Recruiting Hepatoma-Derived Growth Factor/c-Jun Signal in Hepatocellular Carcinoma
Source: Front Cell Dev Biol. 2021 Jul 23;9:659680. doi: 10.3389/fcell.2021.659680 (PMC8343235; doi:10.3389/fcell.2021.659680)
Supplement: Supplementary file 7 [file Table_4.doc]

Supplyment Tab.4 Abbreviation index

| Abbreviation | Full name |
| --- | --- |
| CCND1 | Cyclin D1 |
| c-Jun | 窗体顶端  c-Jun proto-oncogene |
| Co-IP | Co-Immunoprecipitation |
| DMEM | Dulbecco’s modified Eagle medium |
| EdU | 5-Ethynyl-2'-deoxyuridine |
| FBS | Fatal Bovine Serum |
| HBV | hepatitis B virus |
| HCC | hepatocellular carcinoma |
| HDGF | hepatoma-derived growth factor |
| IF | immune fluorescence |
| IHC | Immunohistochemistry |
| MTT | 3-(4,5)-dimethylthiahiazo (-z-y1)-3,5-di- phenytetrazoliumromide |
| NAP1L1 | nucleosome assembly protein 1-like 1 |
| PRDM8 | PRDI-BF1 and RIZ homology domain containing 8 |
| qPCR | quantitative polymerase chain reaction |
| TCGA | The Cancer Genome Atlas database |
| TMA | Tissue Micriarray |
| WB | Western blot |
